# Maternal preconception thyroid autoimmunity is associated with neonatal birth weight conceived by PCOS women undergoing their first in vitro fertilization/intracytoplasmic sperm injection

**DOI:** 10.1186/s13048-023-01208-z

**Published:** 2023-07-14

**Authors:** Huahua Jiang, Lixue Chen, Ning Huang, Huifeng Shi, Hongbin Chi, Rui Yang, Xiaoyu Long, Jie Qiao

**Affiliations:** 1 Center for Reproductive Medicine, Department of Obstetrics and Gynecology, Peking University Third Hospital, Beijing, 100191 China; 2 National Clinical Research Center for Obstetrics and Gynecology, Peking University Third Hospital, Beijing, China; 3 Key Laboratory of Assisted Reproduction, Peking University, Ministry of Education, Beijing, China; 4 Beijing Key Laboratory of Reproductive Endocrinology and Assisted Reproductive Technology, Beijing, China; 5 Department of Obstetrics and Gynecology, Peking University Third Hospital, Beijing, China; 6 National Centre for Healthcare Quality Management in Obstetrics, Beijing, China; 7 Beijing Advanced Innovation Center for Genomics, Peking University, Beijing, China; 8 Peking-Tsinghua Center for Life Sciences, Peking University, Beijing, China

**Keywords:** Thyroid autoimmunity, PCOS, Birth weight, Preconception, In vitro fertilization

## Abstract

**Background:**

Thyroid autoimmunity and polycystic ovary syndrome (PCOS) are the most common endocrinopathies and have close relationships based on common etiology and pathogenesis, including genetic susceptibility, metabolic disorders, hormonal dysregulation, immune response, and inflammatory activation. The co-occurrence of both diseases is associated with adverse reproductive outcomes, but its effect on neonatal outcomes remains largely unknown. We aim to explore the effect of thyroid autoimmunity on neonatal birth weight in PCOS women undergoing IVF/ICSI.

**Methods:**

This is a retrospective analysis of 486 PCOS women who underwent the first IVF/ICSI cycles and gave birth to 361 singletons and 125 twins during 2018 – 2020 at a reproductive center. The associations between maternal preconception serum thyroid function and autoimmunity indicators and birth weights of the singleton and twin groups were evaluated using generalized linear models (GLMs) and generalized estimate equations (GEEs), respectively. Analyses were further stratified by neonatal sex, maternal age, and maternal preconception BMI to assess the possible interaction effects.

**Results:**

Maternal preconception serum TPOAb had a significant negative association with singleton birth weight (*P* for trends = 0.03). Compared with women in the first tertile of TPOAb, women in the third tertile had a change in singleton birth weight of − 119.72 g (95% CI: − 222.68 g, − 16.70 g). Maternal preconception serum TPOAb had a significant positive association with twin birth weight (*P* for trends = 0.01). Compared with women in the first tertile of TPOAb, women in the third tertile had a change in twin birth weight of 138.62 g (95% CI: 33.96 g, 243.30 g). Besides, maternal preconception serum TPOAb had a specific association with increased twin birth weight for female neonates, a specific association with decreased singleton birth weight for PCOS women under 35 years, and a specific association with decreased twin birth weight for overweight PCOS women (all *P* for interactions < 0.05).

**Conclusions:**

Maternal preconception thyroid autoimmunity may affect the birth weights of both singleton and twin neonates. Further large cohorts and experimental studies are required to confirm these findings and explore the underlying mechanisms.

**Supplementary Information:**

The online version contains supplementary material available at 10.1186/s13048-023-01208-z.

## Background

Polycystic ovary syndrome (PCOS) and autoimmune thyroid disease (AITD), are both commonly occurring endocrinopathies in women of reproductive age since the close correlation between the hypothalamic-pituitary-ovarian (HPO) axis and hypothalamic-pituitary-thyroid (HPT) axis [1–3]. The prevalence of AITD in PCOS women is 18 – 40% according to different ethnicity and PCOS diagnostic criteria, and approximately three-fold compared with those without PCOS [3–6]. Increasing evidence has suggested that Hashimoto’s thyroiditis (HT), as the principal AITD, interacts with PCOS due to genetic- and autoimmunity-related factors, including the role of polymorphisms in susceptibility genes, transforming growth factor beta (TGFβ), regulatory T cells (Tregs), vitamin D deficiency, and sex hormone dysregulation [1, 2].

Both AITD and PCOS can result in a wide range of metabolic syndrome features, including impaired glucose tolerance, insulin resistance, and dyslipidemia, which can result in diabetes, obesity, and cardiovascular disease over a lifetime [7–12]. It has been demonstrated that these two diseases can lead to subfecundity, infertility, pregnancy complications, and even intergenerational risks to children [13, 14]. Moreover, the combined occurrence of AITD and PCOS is associated with a higher risk of metabolic and reproductive complications than either AITD or PCOS alone [15, 16]. Moreover, AITD is related to poor treatment response among infertile PCOS women [17]. Some studies have explored the effect of thyroid dysfunction on pregnancy and neonatal outcomes in PCOS women. A study from Austrian reported that neonates of PCOS women with hypothyroidism had significantly higher thyroid stimulating hormone (TSH) levels than neonates of those without hypothyroidism [18]. A post hoc analysis of two randomized controlled trials from Sweden found that a slighter decrease of serum-free thyroxin (FT4) in PCOS women is associated with lower gestational weight gain and lower risk for gestational diabetes (GDM) [19]. However, there are few studies on the effect of thyroid autoimmunity on neonatal outcomes in PCOS women, and the previous studies mainly concentrated on thyroid function and/or thyroid autoimmunity during pregnancy. The preconception period is critical for the growth and maturation of oocytes. Preconception thyroid dysfunction is likely to cause impaired oocytogenesis, thus leading to subsequent abnormal embryogenesis and adverse neonatal outcomes. A Polish study observed a negative association between serum thyroid peroxidase antibodies (TPOAb) and serum anti-mullerian hormone (AMH) in PCOS women, suggesting an adverse effect of thyroid autoimmunity on ovarian reserve [16]. Another two studies from China focused on PCOS women undergoing in vitro fertilization/intracytoplasmic sperm injection (IVF/ICSI). They found the impact of TSH on oocyte maturation, fertilization, and embryo quality [20, 21]. However, no study has assessed the effect of thyroid function or autoimmunity indicators on neonatal outcomes in PCOS women undergoing assisted reproduction technology (ART) treatment.

This study aims to clarify the effects of maternal preconception serum thyroid function and autoimmunity indicators on the birth weight of neonates conceived by PCOS women undergoing IVF/ICSI treatment.

## Material and methods

### Ethics approval

The study was approved by the institutional ethics board of Peking University Third Hospital (No. 2021SZ – 011), and all participants signed written consent forms.

### Study population

We retrospectively involved participants from the Reproductive Center of Peking University Third Hospital from January 1, 2018, to December 30, 2020. PCOS women aged between 20 – 45 years who underwent their first fresh IVF/ICSI cycles with autologous oocytes were informed of the purpose of our study. According to the Rotterdam criteria, PCOS is diagnosed by at least two of the three symptoms: hyperandrogenism (clinical or biochemical), oligo- or anovulation, and/or polycystic ovary morphology [22]. The exclusion criteria were as follows: 1) history of thyroid disease, thyroid hormone/antithyroid medication, or thyroid surgery (*n* = 15), 2) history of autoimmune disease or recurrent spontaneous abortion (*n* = 1), 3) history of iatrogenic ovarian injury (*n* = 1); 4) chromosomal abnormalities in either of the spouses (*n* = 19); 5) receiving in vitro maturation (IVM) or preimplantation genetic testing (PGT) (*n* = 27); (6) missing information (*n* = 202). Finally, a total of 1550 participants were involved in this study.

After cases of pregnancy failure and triplets were excluded (*n* = 1064), 486 women who successfully gave birth to a total of 361 singletons and 125 twins were included in the current analysis. The baseline reproductive characteristics, cycle characteristics, and birth outcomes (i.e., birth weight, gestational age, neonatal sex, and delivery mode) were extracted from electronic medical records. According to the American College of Obstetricians and Gynecologists (ACOG) guidelines for IVF/ICSI-based conception, the gestational age was calculated as (outcome date – date of transfer) + 14 + development days of the transferred embryo [23].

### Ovarian stimulation and IVF procedures

Patients in this study received a standardized individualized controlled ovarian stimulation (COS) protocol according to ovarian reserve and other characteristics. All enrolled patients received GnRH agonist, GnRH antagonist, or minimal stimulation with clomiphene or letrozole [24]. When at least two dominant follicles reached ≥ 18 mm in diameter, ovulation was induced by injection of 5000 – 10,000 IU hCG (Livzon). Oocytes retrieval was performed transvaginally 36 ± 2 h after hCG administration. According to sperm quality, fertilization was performed by either conventional insemination or ICSI at 4 to 6 h after oocyte retrieval. Up to two day 3 embryos or day 5 – 6 blastocysts were transferred according to the Code of Practice for Assisted Reproductive Technology developed by the Ministry of Health of the People’s Republic of China by specified gynecologists with the same standard protocol.

Luteal-phase support was initiated on the day of oocyte retrieval with oral dydrogesterone (Duphaston, Abbott) at a daily dose of 40 mg or vaginal progesterone (Crinone, Merck Serono) at a daily dose of 90 mg until 10 weeks after conception.

### Pregnancy and neonatal follow-up

The reproductive outcome was measured 14 days after embryo transfer as a positive serum hCG, then confirmed by transvaginal ultrasound 3 – 4 weeks after embryo transfer. Clinical pregnancy was confirmed if gestational sacs with fetal cardiac activity could be observed. Live birth is defined as the delivery of a living fetus beyond 28 weeks of gestation. Experienced follow-up staff recorded information regarding clinical pregnancy, live birth, gestational age, delivery mode, neonatal sex, and neonatal birth weight separately from telephone interviews after delivery.

### Biochemical measures

Serum total thyroxin (T4), FT4, TSH, TPOAbs, and thyroglobulin antibodies (TGAbs) levels were determined by automatic chemiluminescence immunoassays (ADVIA Centaur XP, Siemens Healthcare Diagnostics) within six months before the initiation of COS. According to the manufacturer’s instructions, the clinical reference lines of positive TGAb and TPOAb were > 60 U/mL in concentration. Technicians in the laboratory were blinded to other information about the study population.

### Statistical analysis

Statistical analyses were performed with R (R version 4.0.3). Descriptive statistics were conducted to analyze the clinical characteristics and represented as median (interquartile range [IQR]) or n (%) when appropriate. The differences in clinical characteristics between the singleton and twin groups were analyzed by chi-square or Fisher’s exact test for categorical variables and Kruskal–Wallis tests for continuous variables. Generalized linear models (GLMs) were performed to investigate the associations between maternal preconception serum thyroid function and autoimmunity indicators and birth weight in the singleton group. Generalized estimate equations (GEEs) were performed to investigate the associations between maternal preconception serum thyroid function and autoimmunity indicators and birth weight in the twin group. The normal distribution and identity link were applied for neonatal birth weight. The linear trend tests across tertiles were conducted using an ordinal number (1, 2, and 3).

The potential covariates were chosen if their associations with either birth outcomes or thyroid function were reported [25, 26], or when the effect estimate led to a > 10% change in models to evaluate the effects of thyroid function and autoimmunity indicators on neonatal birth weight [27]. Finally, we retained the following covariates in the models: maternal age and maternal preconception BMI were examined as continuous variables; gestational age (< 37 and 37 – 42 weeks), delivery mode (vaginal delivery and cesarean), and neonatal sex (male and female) were examined as dichotomous variables. All the models were adjusted for the same set of covariates for consistency.

To better interpret the results, the total study population's results were also presented as marginal means adjusted for covariates using the R package “emmeans” (version 1.6.2 – 1). Analyses were further stratified by neonatal sex (male vs. female), maternal age (< 35 vs. ≥ 35 years), and maternal preconception BMI (< 25 vs. ≥ 25 kg/m^2^) to evaluate the possible modification effect. To test the robustness of our results, three sensitivity analyses were conducted as follows: 1) restricting the analyses to women with BMI ≥ 18.5 kg/m^2^; 2) restricting the analyses to women diagnosed with primary infertility; 3) only analyzing women with normal testosterone; 4) only analyzing women who had Day 3 embryo transfer. *P*-values < 0.05 was considered to be statistically significant.

## Results

### Characteristics of the study population

The clinical characteristics of 361 mothers with singletons and 125 mothers with twins are presented in Table 1. Among mothers with singletons and twins, the median maternal ages were 31.0 and 30.0 years, respectively; and the median maternal preconception BMIs were 23.9 and 24.0 kg/m^2^, respectively. Women with singletons had a median preconception serum T4, FT4, TSH, TGAb, and TPOAb with 7.9 μg/dL, 1.3 ng/dL, 2.1 μIU/mL, 15.6 U/mL, and 30.0 U/mL, respectively. While women with twins had a median preconception serum T4, FT4, TSH, TGAb, and TPOAb with 8.2 μg/dL, 1.3 ng/dL, 2.1 μIU/mL, 16.0 U/mL, and 29.4 U/mL, respectively. 14.7% and 16.0% of the PCOS women had positive TGAb in the singleton and twin groups, respectively. 11.6% and 12.0% of the PCOS women had positive TPOAb in the singleton and twin groups, respectively. Most of the participants diagnosed with primary infertility (72.9% of the singleton group, 81.6% of the twin group), applied GnRH antagonist protocol for COS (75.9% of the singleton group, 72.0% of the twin group), and underwent conventional insemination (73.4% of the singleton group, 68.8% of the twin group). Almost all of them had embryo transfers three days after oocyte retrieval (95.3% of the singleton group, 99.2% of the twin group) with two embryos (86.4% of the singleton group, 99.2% of the twin group). Significant differences were observed in transferred embryos number, gestational age, delivery mode, and birth weight between the singleton and twin groups (all *P*—values < 0.001).

**Table 1:** Clinical characteristics of the PCOS women with singleton and twin births (Median ± IQR (range) or n (%))

Characteristics		Singletons *N* = 361	Twins *N* = 125	P^a^
Maternal age, years		31.0 (28.0, 33.0)	30.0 (28.0, 32.0)	0.06
Preconception BMI, kg/m^2^		23.9 (21.3, 27.0)	24.0 (21.5, 27.3)	0.59
AFC, n		16.0 (11.0, 21.0)	16.0 (12.0, 22.0)	0.24
Testosterone, nmol/L		0.7 (0.7, 1.1)	0.7 (0.7, 1.0)	0.90
AMH, ng/mL		4.9 (2.9, 7.4)	5.2 (3.5, 8.2)	0.07
Duration of infertility, years		3.0 (2.0, 4.0)	3.0 (2.0, 5.0)	0.81
T4, μg/dL		7.9 (7.0, 9.0)	8.2 (7.2, 9.2)	0.11
FT4, ng/dL		1.3 (1.2, 1.4)	1.3 (1.2, 1.4)	0.18
TSH, μIU/mL		2.1 (1.6, 2.9)	2.1 (1.6, 3.0)	0.74
TGAb, U/mL		15.6 (15.0, 24.6)	16.0 (15.0, 27.5)	0.54
TPOAb, U/mL		30.0 (28.0, 40.7)	29.4 (28.0, 43.5)	0.98
Positive TGAb^b^	No	308 (85.3)	105 (84.0)	0.72
	Yes	53 (14.7)	20 (16.0)	
Positive TPOAb^b^	No	319 (88.4)	110 (88.0)	0.91
	Yes	42 (11.6)	15 (12.0)	
Infertility type	Primary	263 (72.9)	102 (81.6)	0.05
	Secondary	98 (27.1)	23 (18.4)	
Ovarian stimulation regimen	GnRH antagonist	274 (75.9)	90 (72.0)	0.55
	Long GnRHa	80 (22.2)	31 (24.8)	
	Others^bc^	7 (1.9)	4 (3.2)	
Insemination technique	IVF	265 (73.4)	86 (68.8)	0.35
	ICSI	96 (26.6)	39 (31.2)	
Timing of embryo transfer	Day 3	344 (95.3)	124 (99.2)	0.16
	Day 5	12 (3.3)	1 (0.8)	
	Day 6	5 (1.4)	0 (0.0)	
Transferred embryos number, n	1	49 (13.6)	1 (0.8)	< 0.001
	2	312 (86.4)	124 (99.2)	
Gestational age, weeks	< 37	330 (91.4)	63 (50.4)	< 0.001
	37 − 42	31 (8.6)	62 (49.6)	
Delivery mode	Vaginal delivery	165 (45.7)	13 (10.4)	< 0.001
	Caesarean	196 (54.3)	112 (89.6)	
Neonatal sex	Male	177 (49.0)	128 (51.2)	0.62
	Female	184 (51.0)	122 (48.8)	
Birth weight, g	< 2500	17 (4.7)	130 (52.0)	< 0.001
	2500 − 4000	320 (88.6)	120 (48.0)	
	≥ 4000	24 (6.6)	0 (0.0)	

*Abbreviations*: *PCOS* polycystic ovary syndrome, *IQR* interquartile range, *BMI* body mass index, *AFC* antral follicle count, *AMH *anti-Müllerian hormone, *T4* thyroxine, *FT4* free thyroxine, *TSH* thyroid-stimulating hormone, *TGAb* thyroglobulin antibody, *TPOAb* thyroid peroxidase antibody, *GnRH* gonadotropin-releasing hormone, *GnRHa* gonadotropin-releasing hormone agonist, *IVF* in vitro fertilization, *ICSI* intracytoplasmic sperm injection

^a^*P*-values comparing the differences between the singleton group and the twin group

^b^Clinical reference lines of positive TGAb and TPOAb were > 60 U/mL in concentration

^c^Other protocols include the short GnRHa protocol, ultrashort GnRH antagonist protocol, and minimal stimulation protocol clomiphene or letrozole

### Thyroid function and autoimmunity indicators and neonatal birth weight

Table 2 and Table S1 show the associations between maternal preconception thyroid function and autoimmunity indicators and neonatal birth weights of the singleton and twin groups. We observed a nonmonotonic negative relationship between maternal preconception TPOAb and neonatal birth weight in the singleton group (*P* for trends = 0.03); the estimated mean difference in birth weight when the extreme tertiles were compared was − 119.72 g (95% CI: − 222.68 g, − 16.70 g). However, we observed an association between maternal preconception TPOAb and increased neonatal birth weight in the twin group (*P* for trends = 0.01); the estimated mean difference in birth weight when the extreme tertiles were compared was 138.62 g (95% CI: 33.96 g, 243.30 g). We further assessed the associations between maternal preconception thyroid autoimmunity positivity and neonatal birth weights of the singleton and twin groups. However, no significant association was observed between maternal preconception positive TGAb or TPOAb and neonatal birth weight (Table S2).

**Table 2:** Associations between maternal preconception serum thyroid function and autoimmunity indicators and neonatal birth weight among PCOS women undergoing their first IVF/ICSI cycles^a^

Thyroid function and autoimmunity indicators^b^	Change in birth weight (95% CI), g	
	Singletons^c^ *N* = 361	Twins^d^ *N* = 125
T4		
T1	Ref	Ref
T2	− 4.50 (− 113.47, 104.50)	60.15 (− 62.10, 182.40)
T3	− 71.94 (− 184.56, 40.70)	54.47 (− 69.59, 178.50)
P for trend	0.22	0.38
FT4		
T1	Ref	Ref
T2	− 29.07 (− 139.00, 80.80)	− 45.32 (− 163.11, 72.50)
T3	− 29.24 (− 139.00, 80.90)	− 80.30 (− 201.55, 41.00)
P for trend	0.59	0.19
TSH		
T1	Ref	Ref
T2	− 19.22 (− 130.00, 91.90)	1.59 (− 119.50, 122.70)
T3	− 23.46 (− 135.00, 88.20)	17.77 (− 94.40, 130.00)
P for trend	0.68	0.76
TGAb		
T1	Ref	Ref
T2	− 83.39 (− 206.63, 39.80)	126.25 (− 14.25, 266.80)
T3	− 32.44 (− 134.75, 69.90)	81.49 (− 22.79, 185.80)
P for trend	0.48	0.10
TPOAb		
T1	Ref	Ref
T2	4.47 (− 111.15, 120.10)	8.88 (− 133.30, 151.10)
T3	− 119.72 (− 222.68, − 16.70)	138.62 (33.96, 243.30)
P for trend	0.03	0.01

^a^Adjusted for maternal age (continuous), preconception BMI (continuous), gestational age, delivery mode, and neonatal sex

^b^For singleton pregnancy, the tertiles of T4 are 7.30 and 8.60 μg/dL; the tertiles of FT4 are 1.22 and 1.33 μg/dL; the tertiles of FSH are 1.69 and 2.62 μIU/mL; the tertiles of TGAb are 15.00 and 20.70 U/mL; the tertiles of TPOAb are 28.00 and 37.20 U/mL. For twin pregnancy, the tertiles of T4 are 7.80 and 8.70 μg/dL; the tertiles of FT4 are 1.24 and 1.35 μg/dL; the tertiles of FSH are 1.74 and 2.64 μIU/mL; the tertiles of TGAb are 15.00 and 22.70 U/mL; the tertiles of TPOAb are 28.00 and 38.20 U/mL

^c^Based on the generalized linear model

^d^Based on the generalized estimating equation

### Stratified analyses

**Fig. 1:**
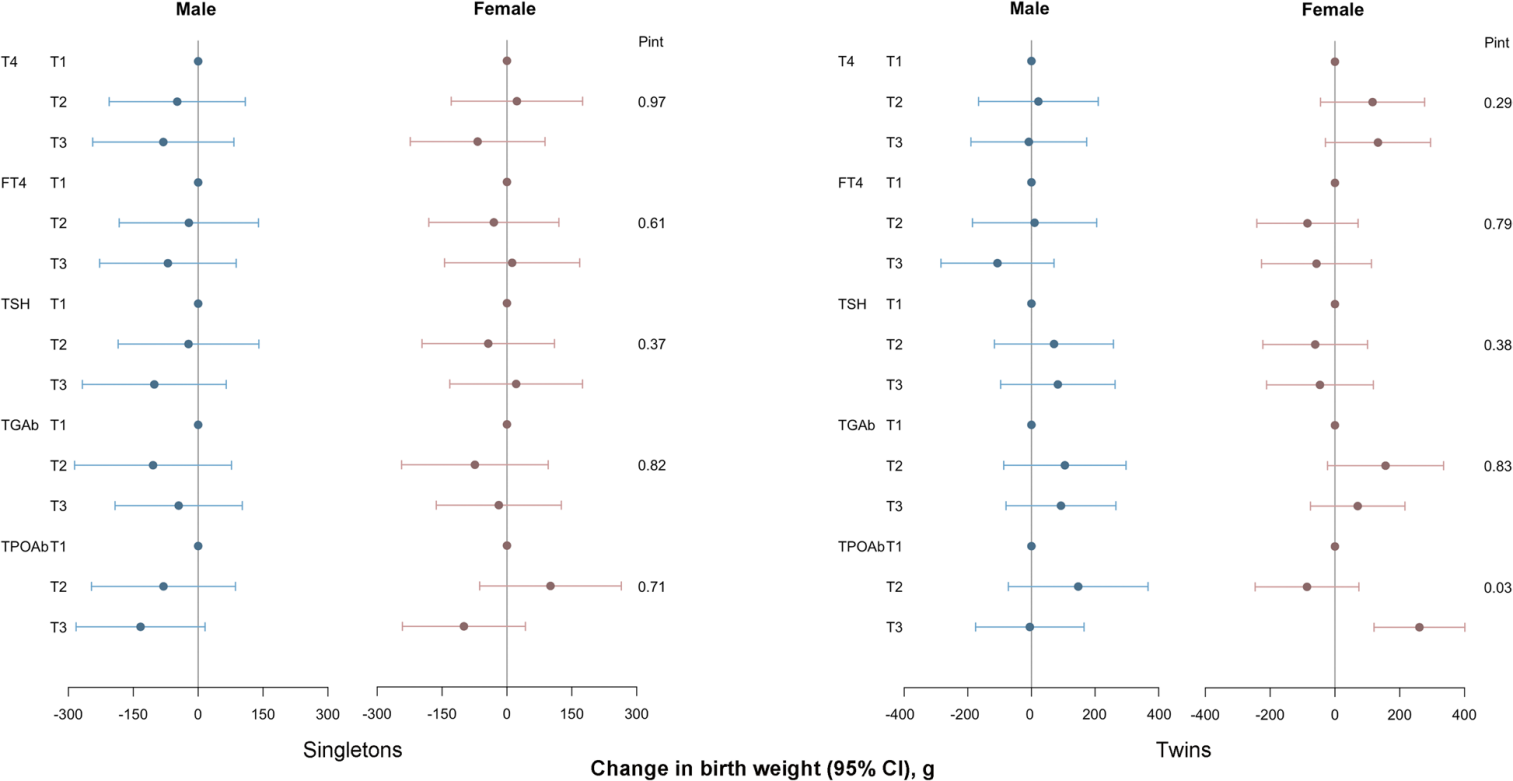
Associations between maternal preconception serum thyroid function and autoimmunity indicators and neonatal birth weight among PCOS women undergoing their first IVF/ICSI cycles stratified by neonatal sex (male vs. female). Adjusted for maternal age (continuous), preconception BMI (continuous), gestational age, and delivery mode. Analyses of singletons and twins were based on the generalized linear model and generalized estimating equation, respectively

**Fig. 2:**
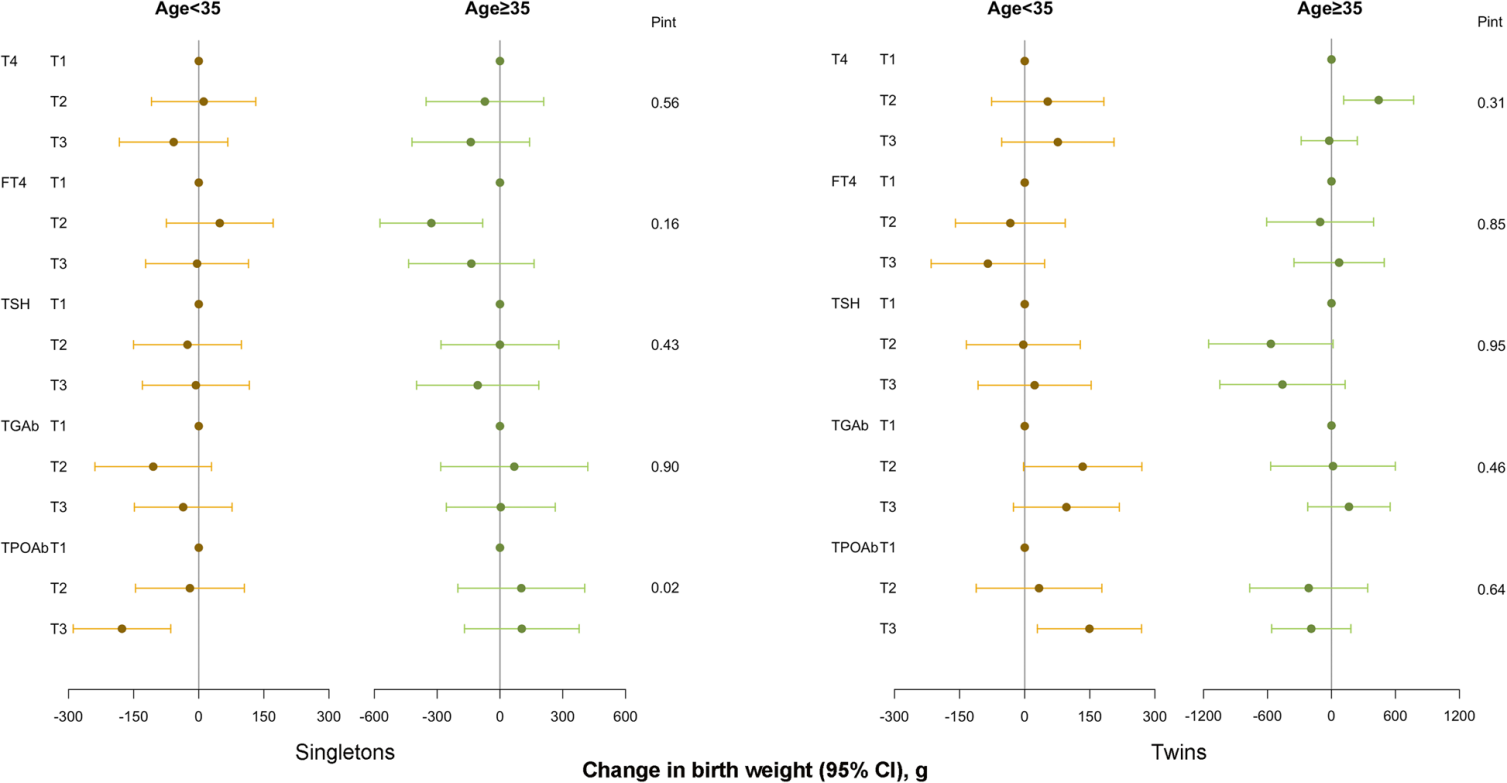
Associations between maternal preconception serum thyroid function and autoimmunity indicators and neonatal birth weight among PCOS women undergoing their first IVF/ICSI cycles stratified by age (< 35 vs. ≥ 35 years). Adjusted for preconception BMI (continuous), gestational age, delivery mode, and neonatal sex. Analyses of singletons and twins were based on the generalized linear model and generalized estimating equation, respectively

**Fig. 3:**
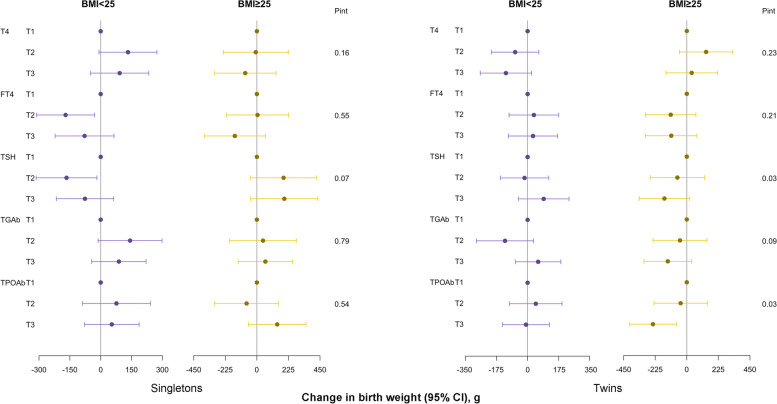
Associations between maternal preconception serum thyroid function and autoimmunity indicators and neonatal birth weight among PCOS women undergoing their first IVF/ICSI cycles stratified by preconception BMI (< 25 vs. ≥ 25 kg/m^2^). Adjusted for maternal age (continuous), gestational age, delivery mode, and neonatal sex. Analyses of singletons and twins were based on the generalized linear model and generalized estimating equation, respectively

The associations between maternal preconception thyroid function and autoimmunity indicators and neonatal birth weights of the singleton and twin groups stratified by neonatal sex are presented in Fig. 1. We found a female-specific association between maternal preconception TPOAb and increased twin birth weight (*P* for interactions = 0.03). The associations between maternal preconception thyroid function and autoimmunity indicators and neonatal birth weights of the singleton and twin groups stratified by maternal age are presented in Fig. 2. We found a significant association between maternal preconception TPOAb and decreased singleton birth weight in PCOS women within 35 years (*P* for interactions = 0.02). The associations between maternal preconception thyroid function and autoimmunity indicators and neonatal birth weights of the singleton and twin groups stratified by maternal preconception BMI are presented in Fig. 3. We found a significant association between maternal preconception TPOAb and decreased twin birth weight in PCOS women with BMI ≥ 25 kg/m^2^ (*P* for interactions = 0.03).

### Sensitivity analyses

Although some significant effects were attenuated, the aforementioned significant associations between maternal preconception TPOAb and offspring birth weights of the singleton and twin groups remained largely unchanged after the analysis was restricted to women with BMI ≥ 18.5 kg/m^2^ (Table S3), women diagnosed with primary infertility (Table S4), women with normal testosterone (Table S5), or women who had Day 3 embryo transfer (Table S6).

## Discussion

Among the PCOS women undergoing the first fresh IVF/ICSI cycles, we found a negative association between maternal preconception TPOAb and singleton birth weight and a positive association between maternal preconception TPOAb and twin birth weight. We found a female-specific positive association between maternal preconception TPOAb and twin birth weight. We also found an age-specific negative association between maternal preconception TPOAb and singleton birth weight among PCOS women within 35 years and a BMI-specific negative association between maternal preconception TPOAb and twin birth weight among PCOS women beyond 25 kg/m^2^.

Thyroid autoimmunity has been proven to be correlated with adverse outcomes during all trimesters of pregnancy [28–30]. It is the leading cause of hypothyroidism among women of reproductive age [31], which itself can negatively affect the whole course of pregnancy [31, 32]. Available evidence shows the association between thyroid autoimmunity, particularly the presence of TPOAb, and poor obstetric and fetal outcomes (i.e., preterm birth, fetal distress, abnormal birth weight, and neurodevelopmental sequelae in children) [30, 33–38]. A meta-analysis of 19 prospective studies included a total of 47 045 women and found that positive TPOAb, subclinical hypothyroidism, and isolated hypothyroxinemia were each associated with a higher risk of preterm birth [36]. Studies found that maternal thyroid autoimmunity during pregnancy was associated with increased NICU admission [39], increased risk for adverse non-verbal cognitive development from infancy to childhood [30], decreased offspring perceptual performance and motor ability [30], and lower Child IQ [40]. A prospective cohort study from Finland reported an adverse effect of maternal TPOAb on intrauterine growth restriction [41]. Nevertheless, this has not been proved via meta-analysis [42]. Birth weight is a simple but important indicator of fetal development and health. A Chinese prospective cohort study found that singletons of clinical hypothyroidism mothers were at a higher risk of low birth weight, whereas it did not specify the levels of thyroid autoantibodies [38]. A Greek prospective cohort study observed a three-fold increased risk for low neonatal birth weight in singletons among women with high TSH and thyroid autoimmunity [43]. However, data are less consistent considering some studies that found no association between hypothyroidism and birthweight with no specification of thyroid autoantibody levels [44–46]. Therefore, more studies are required to confirm the effect of maternal thyroid autoantibody on neonatal outcomes. Interestingly, studies exhibited interactions between thyroid autoimmunity and neonatal sex, suggesting that the correlations between thyroid autoimmunity and neonatal outcomes were adjusted by neonatal sex [35].

Of concern is that maternal preconception thyroid autoimmunity may affect oocytogenesis and subsequent embryogenesis due to the autoimmune activity and cytotoxic reactions [31, 47]. Besides, maternal preconception thyroid autoimmunity may aggravate the imbalance of thyroid function during COS in ART treatment by elevated estradiol level and increased strain on the HPT axis [48]. Thyroid dysfunction may be further aggravated during pregnancy due to the thyroid response mediated by elevated human chorionic gonadotropin (hCG), increased demand for placental transfer of maternal thyroid hormones, the ability of TPOAb to diffuse through the placental barrier, inflammatory reaction, and immune activation [14, 31, 32, 49, 50]. Hence, the preconception period is a potential critical window to assess the effect of thyroid function and/or thyroid autoimmunity on the offspring. Up to now, two studies have explored the associations between preconception thyroid autoimmunity and neonatal birth weight. In a retrospective cohort, the investigators observed increased twin birth weight among diminished ovarian reserve (DOR) women following IVF/ICSI with positive thyroid autoimmunity, whereas the study had a small sample size with only 12 women diagnosed as DOR [51]. Another retrospective study on 778 women undergoing IVF/ICSI with positive thyroid autoimmunity and 778 age-matched control found that thyroid autoimmunity positivity was associated with lower twin birth weight but had no association with singleton birth weight [33]. We focused on the relationship between preconception thyroid autoimmunity and neonatal birth weight in both singleton and twin pregnancies among PCOS women undergoing IVF/ICSI. Specifically, neonatal birth weight from our study had a negative association with maternal preconception TPOAb in the singleton group and a positive association with maternal preconception TPOAb in the twin group. The discrepancies in assays and cutoffs may contribute to the inconsistent results among these studies. Differences in the study populations may also be a possible reason, as the ovaries of women with ovarian dysfunction, such as DOR and PCOS, may be more sensitive to thyroid autoimmunity via complicated pathogenic mechanisms.

The possible cross-mechanisms and potential common genetic backgrounds underlying the close association between AITD and PCOS have been explored in some studies [1, 2]. The joint occurrence of AITD and PCOS was found to increase the risk of dyslipidemia [12], which might further aggravate hypothyroidism via increased insulin resistance and secretion of proinflammatory mediators [52]. Vitamin D deficiency is another possible link between thyroid autoimmunity and PCOS. The decreased vitamin D levels caused by the VDR gene polymorphisms are associated with HT and several features of metabolic syndrome in PCOS women [1]. Moreover, studies suggest that sex hormone dysregulation in PCOS may promote the change of fetus thymus and predispose excessive inflammatory response, thus further leading to autoimmune disorders such as dysregulation of Treg cells [2]. Some evidence has shown abnormally elevated serum anti-histone and anti-double stranded DNA (anti-dsDNA) in PCOS women, which are considered classic markers of autoimmune disease, suggesting an autoimmune background of PCOS [53]. Considering the possible common genetic background, several candidate gene polymorphisms have been proposed to be causally associated with the joint occurrence of HT and PCOS, including polymorphisms in *CYP1B1*, a gene involved in estradiol metabolism; *GNRHR,* a gene related to functions of GnRH; and *FBN3*, a gene linked to TGF-β activity and Treg cell levels [2]. Besides, epigenetic regulation represented as non-coding RNA (ncRNA) may be involved in the joint occurrence of HT and PCOS by modulating the TGF-β signaling pathway [2]. In brief, the association between AITD and PCOS may be ascribed to the potential interaction among genetic susceptibility, metabolic imbalance, hormonal dysregulation, immune response, and inflammatory activation. Although these findings require confirmation in further studies, the close multidirectional mechanistic correlations between AITD and PCOS will help to interpret our results better.

In our study, preconception TPOAb in PCOS women was associated with increased twin birth weight, suggesting that TPOAb may induce abnormal lipid accumulation in twin neonates. Thyroid autoimmunity has been proven to increase the risk of dyslipidemia and subsequent activation of the inflammatory response in PCOS women [12]. This could be aggravated in twin pregnancy, which is under a higher risk of thyroid dysfunction due to increased thyroid hormone demand and stronger thyroid response mediated via higher hCG level as compared with singleton pregnancy [54]. Evidence showed more active esters synthesis on the fetal side of the placenta of twins than singletons, indicating a more significant transfer of lipids to the fetal circulation in twin pregnancy [55]. Moreover, preconception TPOAb in PCOS women was hypothesized to cause adverse singleton birth weight via oocyte and embryo toxicity caused by autoimmune activity and cytotoxic responses in our study [31]. Thyroid hormone transporters, thyroid hormone receptors, and deiodinases can express in the ovarian follicle and pre-implantation embryo [31, 47, 56]. And thyroid autoimmunity has been elucidated to have adverse effects on the number of oocytes retrieved and embryo development outcomes among women undergoing ART [31, 33]. Besides, the clinical baseline characteristics of our study population indicated that twin neonates had significantly lower birth weights than singleton neonates. Thus, the difference in the impact of thyroid autoimmunity on the birth weights of the singleton and twin groups may be related to the difference in the weight ranges of the two groups, suggesting the possibility of a potential nonlinear relationship between thyroid autoimmunity and birth weight. Of note is that we found no effect of positive TGAb or TPOAb on singleton and twin birth weights. This may be due to the bias caused by the low proportion of PCOS women with positive thyroid autoimmunity in the total study population of this study.

Studies have exhibited interactions between thyroid autoimmunity and neonatal sex [35]. In this study, we found that maternal preconception TPOAb is associated with increased birth weight in female twins. Female neonates may have higher risks of abnormal immune and metabolic functions due to the close relationship between HPO and HPT axes [57], thus leading to abnormal lipid accumulation. We also found maternal preconception TPOAb is associated with adverse singleton birth weight in PCOS women within 35 years and adverse twin birth weight in PCOS women beyond 25 kg/m^2^. The relatively high estrogen levels of young women and abnormal lipid metabolism in overweight women may further aggravate thyroid autoimmune abnormalities [2], damage oocytes and embryos, and result in adverse neonatal outcomes. Besides, our results suggest that age is more important than thyroid autoimmunity in predicting neonatal birth weight in advanced-aged PCOS women.

To our knowledge, this is the first study on the effect of maternal preconception thyroid autoimmunity on neonatal birth weight among PCOS women undergoing IVF/ICSI. However, some limitations of our study must be considered. First, it is a retrospective and observational cohort study, and bias in data collection may cause erroneous data interpretation. However, thyroid function and autoimmunity indicators, neonatal birth weight, and covariates were all objective indicators, thus can eliminate the bias to some extent. Second, we did not consider the thyroid function and autoimmunity indicators during pregnancy, a period of dramatic change in thyroid function and/or thyroid autoimmunity [32, 50]. Thus, further studies focused on preconception and all trimesters of pregnancy will provide more solid evidence. Third, while many confounders have been adjusted in our model, other potential confounders cannot be ignored. However, we have appropriately screened covariates based on previous literature and appropriate principles [27]. Fourth, we did not consider PCOS women undergoing frozen-thawed embryo transfer (FET). However, due to the long treatment period, the thyroid function, thyroid autoimmunity, and birth outcomes of PCOS women undergoing FET may be affected by more complex confounding factors, which may add to the bias of this study. Finally, we did not consider the use of levothyroxine due to the retrospective design, whereas our previous randomized clinical trial reported no effect of levothyroxine on pregnancy outcomes [24]. More studies are further required to focus on levothyroxine's influence on neonatal outcomes.

## Conclusion

In conclusion, we found that maternal preconception TPOAb was negatively associated with singleton birth weight and positively associated with twin birth weight among PCOS women undergoing IVF/ICSI. Abnormal maternal thyroid autoimmunity may lead to neonatal lipid accumulation in PCOS women with twin pregnancies. It may also cause adverse neonatal outcomes through oocyte and embryo toxicity. While the results of the present study need to be further confirmed since the lack of well-established relevant research, our findings suggest that the preconception thyroid autoimmunity may be crucial for offspring growth and development.

## Supplementary Information

**Additional file 1: Table S1.** Adjusted mean (95% CI) for neonatal birth weight by maternal preconception serum thyroid function and autoimmunity indicators among PCOS women undergoing their first IVF/ICSI cycles^a^.

**Additional file 2: Table S2.** Associations between maternal preconception thyroid autoimmunity positivity and neonatal birth weight among PCOS women undergoing their first IVF/ICSI cycles^a^.

**Additional file 3: Table S3.** Associations between maternal preconception serum thyroid function and autoimmunity indicators and neonatal birth weight among PCOS women with preconception BMI ≥18.5kg/m^2^ undergoing their first IVF/ICSI cycles^a^.

**Additional file 4: Table S4.** Associations between maternal preconception serum thyroid function and autoimmunity indicators and neonatal birth weight among PCOS women with primary infertility undergoing their first IVF/ICSI cycles^a^.

**Additional file 5: Table S5.** Associations between t maternal preconception serum thyroid function and autoimmunity indicators and neonatal birth weight among PCOS women with normal testosterone undergoing their first IVF/ICSI cycles^a^.

**Additional file 6: Table S6.** Associations between maternal preconception serum thyroid function and autoimmunity indicators and neonatal birth weight among PCOS women undergoing their first IVF/ICSI cycles with Day 3 embryos transferred^a^.
