# Supplementary material for: Maternal preconception thyroid autoimmunity is associated with neonatal birth weight conceived by PCOS women undergoing their first in vitro fertilization/intracytoplasmic sperm injection
Source: J Ovarian Res. 2023 Jul 14;16:140. doi: 10.1186/s13048-023-01208-z (PMC10347740; doi:10.1186/s13048-023-01208-z)
Supplement: Supplementary file 1 — Additional file 1: Table S1. Adjusted mean (95% CI) for neonatal birth weight by maternal preconception serum thyroid function and autoimmunity indicators among PCOS women undergoing their first IVF/ICSI cyclesa. [file 13048_2023_1208_MOESM1_ESM.docx]

| **Table S1.** Adjusted mean (95% CI) for neonatal birth weight by maternal preconception serum thyroid function and autoimmunity indicators among PCOS women undergoing their first IVF/ICSI cycles^a^. | | |
| --- | --- | --- |
| **Thyroid function and autoimmunity indicators**^b^ | **Adjusted mean birth weight (95% CI), g** | |
|  | **Singletons**^c^  **N=361** | **Twins**^d^  **N=125** |
| T4 |  |  |
| T1 | 2941 (2840, 3043) | 2308 (2184, 2433) |
| T2 | 2937 (2834, 3039) | 2369 (2251, 2486) |
| T3 | 2869 (2765, 2974) | 2363 (2250, 2476) |
| P for trend | 0.22 | 0.38 |
| FT4 |  |  |
| T1 | 2934 (2836, 3033) | 2389 (2259, 2520) |
| T2 | 2905 (2804, 3006) | 2344 (2215, 2472) |
| T3 | 2905 (2794, 3016) | 2309 (2199, 2418) |
| P for trend | 0.59 | 0.19 |
| TSH |  |  |
| T1 | 2931 (2828, 3034) | 2339 (2229, 2450) |
| T2 | 2912 (2807, 3017) | 2341 (2217, 2464) |
| T3 | 2908 (2806, 3009) | 2357 (2227, 2487) |
| P for trend | 0.68 | 0.76 |
| TGAb |  |  |
| T1 | 2941 (2850, 3031) | 2284 (2170, 2397) |
| T2 | 2857 (2732, 2982) | 2410 (2279, 2541) |
| T3 | 2908 (2803, 3013) | 2365 (2257, 2473) |
| P for trend | 0.48 | 0.10 |
| TPOAb |  |  |
| T1 | 2956 (2863, 3050) | 2300 (2194, 2406) |
| T2 | 2961 (2844, 3078) | 2309 (2160, 2457) |
| T3 | 2837 (2735, 2938) | 2438 (2330, 2547) |
| P for trend | 0.03 | 0.01 |
| ^a^ Adjusted for maternal age (continuous), preconception BMI (continuous), gestational age, delivery mode, and neonatal sex.  ^b^ For singleton pregnancy, the tertiles of T4 are 7.30 and 8.60 μg/dL; the tertiles of FT4 are 1.22 and 1.33 μg/dL; the tertiles of FSH are 1.69 and 2.62 μIU/mL; the tertiles of TGAb are 15.00 and 20.70 U/mL; the tertiles of TPOAb are 28.00 and 37.20 U/mL. For twin pregnancy, the tertiles of T4 are 7.80 and 8.70 μg/dL; the tertiles of FT4 are 1.24 and 1.35 μg/dL; the tertiles of FSH are 1.74 and 2.64 μIU/mL; the tertiles of TGAb are 15.00 and 22.70 U/mL; the tertiles of TPOAb are 28.00 and 38.20 U/mL. ^c^ Based on the generalized linear model.  ^d^ Based on the generalized estimating equation. | | |
