# Supplementary material for: Maternal preconception thyroid autoimmunity is associated with neonatal birth weight conceived by PCOS women undergoing their first in vitro fertilization/intracytoplasmic sperm injection
Source: J Ovarian Res. 2023 Jul 14;16:140. doi: 10.1186/s13048-023-01208-z (PMC10347740; doi:10.1186/s13048-023-01208-z)
Supplement: Supplementary file 2 — Additional file 2: Table S2. Associations between maternal preconception thyroid autoimmunity positivity and neonatal birth weight among PCOS women undergoing their first IVF/ICSI cyclesa. [file 13048_2023_1208_MOESM2_ESM.docx]

| **Table S2.** Associations between maternal preconception thyroid autoimmunity positivity and neonatal birth weight among PCOS women undergoing their first IVF/ICSI cycles^a^. | | | | |  |
| --- | --- | --- | --- | --- | --- |
| **Thyroid autoimmunity positivity**^b^ | | **Change in birth weight (95% CI), g** | | | |
|  |  | **Singletons**^c^  **N=361** | **Twins**^d^  **N=125** | | |
| Positive TGAb |  | | |  |  |
| No | Ref. | | | Ref. |  |
| Yes | 37.74 (−89.50, 165.00) | | | −41.22 (−147.63, 65.20) |  |
| P | 0.56 | | | 0.45 |  |
| Positive TPOAb |  | | |  |  |
| No | Ref. | | | Ref. |  |
| Yes | −27.85 (−168.00, 112.10) | | | 40.90 (−93.2, 174.90) |  |
| P | 0.70 | | | 0.55 |  |
| ^a^ Adjusted for maternal age (continuous), preconception BMI (continuous), gestational age, delivery mode, and neonatal sex.  ^b^ Clinical reference lines of positive TGAb and TPOAb were > 60 U/mL in concentration. ^c^ Based on the generalized linear model.  ^d^ Based on the generalized estimating equation. | | | | |  |
