# Supplementary material for: Maternal preconception thyroid autoimmunity is associated with neonatal birth weight conceived by PCOS women undergoing their first in vitro fertilization/intracytoplasmic sperm injection
Source: J Ovarian Res. 2023 Jul 14;16:140. doi: 10.1186/s13048-023-01208-z (PMC10347740; doi:10.1186/s13048-023-01208-z)
Supplement: Supplementary file 3 — Additional file 3: Table S3. Associations between maternal preconception serum thyroid function and autoimmunity indicators and neonatal birth weight among PCOS women with preconception BMI ≥18.5kg/m2 undergoing their first IVF/ICSI cyclesa. [file 13048_2023_1208_MOESM3_ESM.docx]

| **Table S3.** Associations between maternal preconception serum thyroid function and autoimmunity indicators and neonatal birth weight among PCOS women with preconception BMI ≥18.5kg/m^2^ undergoing their first IVF/ICSI cycles^a^. | | |
| --- | --- | --- |
| **Thyroid function and autoimmunity indicators**^b^ | **Change in birth weight (95% CI), g** | |
|  | **Singletons**^c^  **N=347** | **Twins**^d^  **N=119** |
| T4 |  |  |
| T1 | Ref. | Ref. |
| T2 | 0.57 (−112.72, 113.85) | 70.90 (−53.67, 195.46) |
| T3 | −65.31 (−179.64, 49.01) | 69.85 (−55.19, 194.89) |
| P for trend | 0.26 | 0.27 |
| FT4 |  |  |
| T1 | Ref. | Ref. |
| T2 | −19.85 (−132.58, 92.88) | −30.61 (−154.91, 93.70) |
| T3 | −33.95 (−147.96, 80.05) | −69.57 (−193.78, 54.64) |
| P for trend | 0.56 | 0.27 |
| TSH |  |  |
| T1 | Ref. | Ref. |
| T2 | −24.60 (−139.91, 90.72) | 13.51 (−110.43, 137.44) |
| T3 | −19.87 (−133.78, 94.03) | 35.42 (−90.49, 161.33) |
| P for trend | 0.73 | 0.58 |
| TGAb |  |  |
| T1 | Ref. | Ref. |
| T2 | −100.16 (−227.91, 27.60) | 134.99 (3.97, 266.01) |
| T3 | −29.22 (−134.16, 75.72) | 98.14 (−17.80, 214.08) |
| P for trend | 0.52 | 0.08 |
| TPOAb |  |  |
| T1 | Ref. | Ref. |
| T2 | 16.01 (−108.39, 135.62) | 21.29 (−115.55, 158.12) |
| T3 | −108.39 (−213.94, −2.84) | 168.66 (51.57, 285.75) |
| P for trend | 0.05 | <0.01 |
| ^a^ Adjusted for maternal age (continuous), preconception BMI (continuous), gestational age, delivery mode, and neonatal sex.  ^b^ For singleton pregnancy, the tertiles of T4 are 7.30 and 8.60 μg/dL; the tertiles of FT4 are 1.22 and 1.33 μg/dL; the tertiles of FSH are 1.69 and 2.62 μIU/mL; the tertiles of TGAb are 15.00 and 20.70 U/mL; the tertiles of TPOAb are 28.00 and 37.20 U/mL. For twin pregnancy, the tertiles of T4 are 7.80 and 8.70 μg/dL; the tertiles of FT4 are 1.24 and 1.35 μg/dL; the tertiles of FSH are 1.74 and 2.64 μIU/mL; the tertiles of TGAb are 15.00 and 22.70 U/mL; the tertiles of TPOAb are 28.00 and 38.20 U/mL. ^c^ Based on the generalized linear model.  ^d^ Based on the generalized estimating equation. | | |
