# Supplementary material for: Maternal preconception thyroid autoimmunity is associated with neonatal birth weight conceived by PCOS women undergoing their first in vitro fertilization/intracytoplasmic sperm injection
Source: J Ovarian Res. 2023 Jul 14;16:140. doi: 10.1186/s13048-023-01208-z (PMC10347740; doi:10.1186/s13048-023-01208-z)
Supplement: Supplementary file 4 — Additional file 4: Table S4. Associations between maternal preconception serum thyroid function and autoimmunity indicators and neonatal birth weight among PCOS women with primary infertility undergoing their first IVF/ICSI cyclesa. [file 13048_2023_1208_MOESM4_ESM.docx]

| **Table S4.** Associations between maternal preconception serum thyroid function and autoimmunity indicators and neonatal birth weight among PCOS women with primary infertility undergoing their first IVF/ICSI cycles^a^. | | |
| --- | --- | --- |
| **Thyroid function and autoimmunity indicators**^b^ | **Change in birth weight (95% CI), g** | |
|  | **Singletons**^c^  **N=263** | **Twins**^d^  **N=102** |
| T4 |  |  |
| T1 | Ref. | Ref. |
| T2 | −38.68 (−162.87, 85.52) | 42.50 (−95.69, 180.70) |
| T3 | −131.65 (−256.24, −7.05) | 26.06 (−107.72, 159.84) |
| P for trend | 0.04 | 0.72 |
| FT4 |  |  |
| T1 | Ref. | Ref. |
| T2 | −35.28 (−162.53, 91.97) | −78.02 (−211.40, 55.37) |
| T3 | −73.11 (−194.63, 48.41) | −106.36 (−236.45, 23.74) |
| P for trend | 0.24 | 0.11 |
| TSH |  |  |
| T1 | Ref. | Ref. |
| T2 | −56.83 (−186.01, 72.34) | 60.26 (−73.13, 193.65) |
| T3 | −13.50 (−138.01, 111.01) | 74.08 (−61.91, 210.07) |
| P for trend | 0.86 | 0.29 |
| TGAb |  |  |
| T1 | Ref. | Ref. |
| T2 | −90.77 (−227.46, 45.92) | 90.01 (−54.89, 234.91) |
| T3 | −62.52 (−182.22, 57.17) | 78.38 (−44.74, 201.49) |
| P for trend | 0.26 | 0.19 |
| TPOAb |  |  |
| T1 | Ref. | Ref. |
| T2 | 20.58 (−104.65, 145.81) | −19.25 (−175.95, 137.44) |
| T3 | −164.00 (−281.42, −46.57) | 114.86 (−6.14, 235.86) |
| P for trend | <0.01 | 0.08 |
| ^a^ Adjusted for maternal age (continuous), preconception BMI (continuous), gestational age, delivery mode, and neonatal sex.  ^b^ For singleton pregnancy, the tertiles of T4 are 7.30 and 8.60 μg/dL; the tertiles of FT4 are 1.22 and 1.33 μg/dL; the tertiles of FSH are 1.69 and 2.62 μIU/mL; the tertiles of TGAb are 15.00 and 20.70 U/mL; the tertiles of TPOAb are 28.00 and 37.20 U/mL. For twin pregnancy, the tertiles of T4 are 7.80 and 8.70 μg/dL; the tertiles of FT4 are 1.24 and 1.35 μg/dL; the tertiles of FSH are 1.74 and 2.64 μIU/mL; the tertiles of TGAb are 15.00 and 22.70 U/mL; the tertiles of TPOAb are 28.00 and 38.20 U/mL. ^c^ Based on the generalized linear model.  ^d^ Based on the generalized estimating equation. | | |
