# Supplementary material for: Maternal preconception thyroid autoimmunity is associated with neonatal birth weight conceived by PCOS women undergoing their first in vitro fertilization/intracytoplasmic sperm injection
Source: J Ovarian Res. 2023 Jul 14;16:140. doi: 10.1186/s13048-023-01208-z (PMC10347740; doi:10.1186/s13048-023-01208-z)
Supplement: Supplementary file 5 — Additional file 5: Table S5. Associations between t maternal preconception serum thyroid function and autoimmunity indicators and neonatal birth weight among PCOS women with normal testosterone undergoing their first IVF/ICSI cyclesa. [file 13048_2023_1208_MOESM5_ESM.docx]

| **Table S5.** Associations between t maternal preconception serum thyroid function and autoimmunity indicators and neonatal birth weight among PCOS women with normal testosterone undergoing their first IVF/ICSI cycles^a^. | | |
| --- | --- | --- |
| **Thyroid function and autoimmunity indicators**^b^ | **Change in birth weight (95% CI), g** | |
|  | **Singletons**^c^  **N=356** | **Twins**^d^  **N=121** |
| T4 |  |  |
| T1 | Ref. | Ref. |
| T2 | −13.75 (−123.64, 96.15) | 67.25 (−54.58, 189.08) |
| T3 | −76.56 (−190.51, 37.40) | 69.16 (−53.62, 191.95) |
| P for trend | 0.19 | 0.26 |
| FT4 |  |  |
| T1 | Ref. | Ref. |
| T2 | −33.45 (−144.24, 77.34) | −37.10 (−157.53, 83.34) |
| T3 | −26.81 (−137.76, 84.14) | −72.80 (−196.83, 51.24) |
| P for trend | 0.62 | 0.25 |
| TSH |  |  |
| T1 | Ref. | Ref. |
| T2 | −23.98 (−136.46, 88.50) | −12.10 (−134.87, 110.66) |
| T3 | −25.18 (−137.41, 87.06) | −13.76 (−137.46, 109.94) |
| P for trend | 0.66 | 0.83 |
| TGAb |  |  |
| T1 | Ref. | Ref. |
| T2 | −86.65 (−210.54, 37.24) | 112.29 (−19.70, 244.28) |
| T3 | −40.42 (−144.11, 63.27) | 77.83 (−35.53, 191.18) |
| P for trend | 0.40 | 0.15 |
| TPOAb |  |  |
| T1 | Ref. | Ref. |
| T2 | 9.72 (−106.88, 126.33) | −11.88 (−149.46, 125.70) |
| T3 | −117.17 (−220.99, −13.35) | 137.17 (24.12, 250.21) |
| P for trend | 0.03 | 0.02 |
| ^a^ Adjusted for maternal age (continuous), preconception BMI (continuous), gestational age, delivery mode, and neonatal sex.  ^b^ For singleton pregnancy, the tertiles of T4 are 7.30 and 8.60 μg/dL; the tertiles of FT4 are 1.22 and 1.33 μg/dL; the tertiles of FSH are 1.69 and 2.62 μIU/mL; the tertiles of TGAb are 15.00 and 20.70 U/mL; the tertiles of TPOAb are 28.00 and 37.20 U/mL. For twin pregnancy, the tertiles of T4 are 7.80 and 8.70 μg/dL; the tertiles of FT4 are 1.24 and 1.35 μg/dL; the tertiles of FSH are 1.74 and 2.64 μIU/mL; the tertiles of TGAb are 15.00 and 22.70 U/mL; the tertiles of TPOAb are 28.00 and 38.20 U/mL. ^c^ Based on the generalized linear model.  ^d^ Based on the generalized estimating equation. | | |
