# Supplementary material for: Maternal preconception thyroid autoimmunity is associated with neonatal birth weight conceived by PCOS women undergoing their first in vitro fertilization/intracytoplasmic sperm injection
Source: J Ovarian Res. 2023 Jul 14;16:140. doi: 10.1186/s13048-023-01208-z (PMC10347740; doi:10.1186/s13048-023-01208-z)
Supplement: Supplementary file 6 — Additional file 6: Table S6. Associations between maternal preconception serum thyroid function and autoimmunity indicators and neonatal birth weight among PCOS women undergoing their first IVF/ICSI cycles with Day 3 embryos transferreda. [file 13048_2023_1208_MOESM6_ESM.docx]

| **Table S6.** Associations between maternal preconception serum thyroid function and autoimmunity indicators and neonatal birth weight among PCOS women undergoing their first IVF/ICSI cycles with Day 3 embryos transferred^a^. | | |
| --- | --- | --- |
| **Thyroid function and autoimmunity indicators**^b^ | **Change in birth weight (95% CI), g** | |
|  | **Singletons**^c^  **N=344** | **Twins**^d^  **N=124** |
| T4 |  |  |
| T1 | Ref. | Ref. |
| T2 | −0.17 (−112.63, 112.29) | 59.02 (−64.40, 182.40) |
| T3 | −60.17 (−177.60, 57.26) | 53.28 (−72.29, 178.90) |
| P for trend | 0.33 | 0.40 |
| FT4 |  |  |
| T1 | Ref. | Ref. |
| T2 | −24.62 (−138.99, 89.75) | −48.30 (−167.60, 71.10) |
| T3 | −15.99 (−129.89, 97.91) | −83.20 (−205.79, 39.30) |
| P for trend | 0.77 | 0.18 |
| TSH |  |  |
| T1 | Ref. | Ref. |
| T2 | −19.91 (−135.15, 95.32) | −0.27 (−122.14, 121.60) |
| T3 | −29.32 (−145.87, 87.23) | −15.87 (−97.25, 129.00) |
| P for trend | 0.62 | 0.78 |
| TGAb |  |  |
| T1 | Ref. | Ref. |
| T2 | −82.22 (−211.84, 47.39) | 132.09 (−10.90, 275.10) |
| T3 | −39.16 (−145.45, 67.13) | 81.24 (−23.05, 185.50) |
| P for trend | 0.42 | 0.10 |
| TPOAb |  |  |
| T1 | Ref. | Ref. |
| T2 | 14.11 (−134.57, 106.34) | 11.75 (−135.92, 159.40) |
| T3 | −126.06 (−233.08, −19.04) | 138.79 (34.07, 243.50) |
| P for trend | 0.02 | 0.01 |
| ^a^ Adjusted for maternal age (continuous), preconception BMI (continuous), gestational age, delivery mode, and neonatal sex.  ^b^ For singleton pregnancy, the tertiles of T4 are 7.30 and 8.60 μg/dL; the tertiles of FT4 are 1.22 and 1.33 μg/dL; the tertiles of FSH are 1.69 and 2.62 μIU/mL; the tertiles of TGAb are 15.00 and 20.70 U/mL; the tertiles of TPOAb are 28.00 and 37.20 U/mL. For twin pregnancy, the tertiles of T4 are 7.80 and 8.70 μg/dL; the tertiles of FT4 are 1.24 and 1.35 μg/dL; the tertiles of FSH are 1.74 and 2.64 μIU/mL; the tertiles of TGAb are 15.00 and 22.70 U/mL; the tertiles of TPOAb are 28.00 and 38.20 U/mL. ^c^ Based on the generalized linear model.  ^d^ Based on the generalized estimating equation. | | |
